# Castleman disease: Case series of two surgical patients from different ends of the disease spectrum with literature review

**DOI:** 10.1016/j.ijscr.2018.10.053

**Published:** 2018-10-29

**Authors:** Radhika Raj C.G., Suresh B.

**Affiliations:** Goa Medical College, Bambolim, Goa, 403202, India

**Keywords:** Castleman disease, HHV8, Lymphoproliferative disorder, Cytokine storm, Interleukin-6

## Abstract

Castleman Disease (CD) is a rare, heterogeneous group of hyperimmune lymphoproliferative disorders, not very familiar to surgeons. Unicentric Castleman Disease (UCCD) at one end of the spectrum is a localized disease, with little or no systemic symptoms. It may be an incidental radiological finding or detected while investigating for a symptomatic lymph node mass. Surgery is the primary treatment and has good long term prognosis.

Multicentric Castleman Disease (MCCD) is a more serious systemic condition, often associated with constitutional symptoms. Exaggerated systemic inflammatory response secondary to “Cytokine storm” involving Interleukin-6 (IL-6) may cause multi-organ dysfunction. In addition, immunosuppression or malignant transformation can prove lethal. Human Herpes Simplex Virus 8 (HHV8) associated MCCD is a major subgroup occurring in immunocompromised individuals due to the viral trigger. Antiviral therapy is important in its treatment. Idiopathic MCCD (IMCCD) has no known biomarker and is diagnosed after excluding infective, autoimmune and malignant conditions of lymphoid tissue. IMCCD requires systemic therapy.

We report a patient of UCCD who presented as a retroperitoneal mass in right iliac fossa causing pressure on femoral nerve. Following successful surgical excision she had good recovery. We report another patient who had large inguinal lymph node mass with constitutional symptoms. IMCCD was diagnosed after excision biopsy and comprehensive work up. Patient was started on corticosteroids followed by CD-20 targeted therapy.

These two cases showcase the two ends of the clinical spectrum of CD requiring different management protocols. Awareness among surgeons and diligent work-up is imperative for early diagnosis and best outcome.

## Highlights

• Castleman disease: heterogeneous group of hyperimmune lymphoproliferative disorders.
• Unicentric type: localized disease; surgery is primary treatment; good prognosis.
• Multicentric type: Serious systemic disease; 70% linked to HHV8; antiviral therapy.
• Idiopathic Multicentric type: diagnosis by exclusion; Interleukin-6 blockade useful.

## 1 Introduction

Castleman Disease (CD) is a rare, heterogeneous group of hyperimmune lymphoproliferative disorders described by Benjamin Castleman in 1956. [1] It is also known as giant lymph node hyperplasia or angiofollicular lymph node hyperplasia.

Unicentric Castleman Disease (UCCD) at one end of the spectrum is a localized disease, presenting with enlarged lymph nodes in a single station with little or no systemic symptoms. The disease may be detected incidentally on radiological imaging or detected while investigating for a symptomatic lymph node mass. Surgery is the primary treatment and has good long term prognosis [2].

In contrast, Multicentric Castleman Disease (MCCD) [2] is a serious systemic condition. Patients often have constitutional symptoms like fever, weight loss or debility. It can progress and become lethal due to: exaggerated systemic inflammatory response and multi-organ dysfunction caused by “Cytokine storm”; [3] immunosuppression or malignant transformation. Human Herpes Simplex Virus 8 (HSSV-8) associated MCCD is a major subgroup occurring in immunocompromised individuals due to the viral trigger. Antiviral therapy [4] has a role in its treatment. Idiopathic MCCD (IMCCD) has no known biomarker [3] and is diagnosed after excluding infective, autoimmune and malignant conditions of lymphoid tissue [3]. IMCCD requires systemic therapy.

We are reporting two cases managed by general surgery department in a state level medical college; to showcase the two ends of the clinical spectrum of CD requiring different management protocols. The reported study is in line with Process criteria. [5]

## 2 Case summary

### 2.1 Case:1

**Fig. 1:**
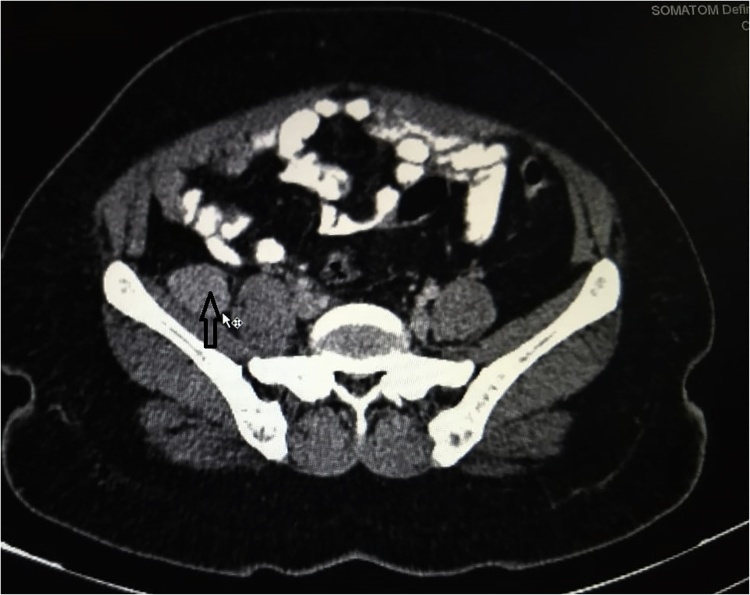
Retroperitoneal mass over right iliacus muscle. Contrast enhanced CT scan of abdomen showing homogeneous, vascular, well defined retroperitoneal solid mass overlying right iliacus muscle (arrow).

A 45 year old lady came with right lower abdominal pain radiating to right lower limb over 4 months. There were no other significant abdominal complaints. Patient did not have any contact with tuberculosis. Clinical examination was normal. Abdominopelvic ultrasonography showed 4 × 3 cm solid mass in right iliac fossa. Contrast enhanced CT of the abdomen (Fig. 1) showed homogeneous, vascular and well defined retroperitoneal solid mass overlying right iliacus muscle; suggestive of a benign neurogenic tumour. At laparotomy, a vascular tumour overlying the right femoral nerve was excised.

**Fig. 2:**
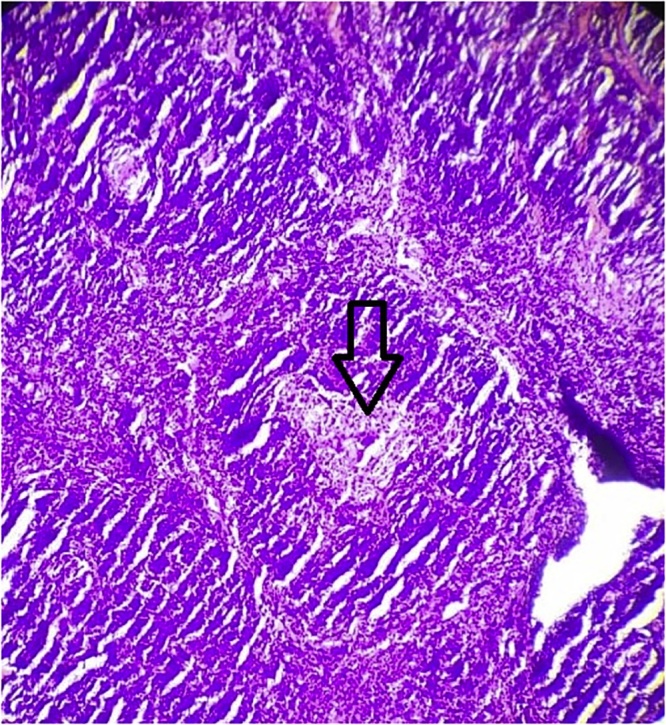
Hyaline vascular variant of Castleman disease. Histopathology shows atrophic germinal centre with vessel traversing through it: “lollypop appearance” (black arrow). The expanded mantle zone shows concentric rings of small lymphocytes: “onion skin appearance”- hyaline vascular variant of Castleman disease.

Histopathology (Fig. 2) showed atrophic germinal centre with vessel traversing through it: “lollypop appearance”. The expanded mantle zone shows concentric rings of small lymphocytes: “onion skin appearance”- suggestive of hyaline vascular variant of Castleman disease.

All biochemical investigations and PET CT were normal. Patient is on follow up for 6 months and is doing well.

### 2.2 Case-2

A 33 year old male came with rapidly growing left inguinal lymph node mass, low-grade fever, loss of weight and severe debility over 2 months. There was no contact with tuberculosis. Examination showed: 6 × 8 cm size painless, firm, left inguinal lymph node mass; small bilateral axillary and cervical lymphadenopathy. Liver and spleen were not palpable. There was pitting edema of left leg.

#### 2.2.1 Investigations

**Fine Needle Aspiration Cytology**- was inconclusive; negative for tuberculous bacilli.

**Fig. 3:**
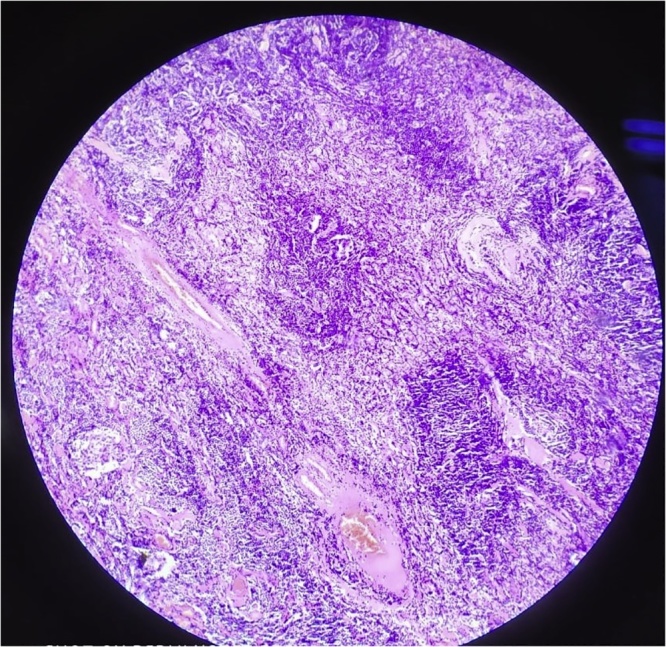
Plasmacytic variant of Castleman disease. Histopathology shows florid reactive follicular pattern with hyperplastic germinal centre, focal inter-follicular predominance of plasma cells and marked vascular proliferation: Plasmacytic variant of Castleman disease.

**Histopathology of excision biopsy specimen** (Fig. 3) showed florid reactive follicular pattern with hyperplastic germinal centre, focal inter-follicular predominance of plasma cells and marked vascular proliferation suggestive of plasmacytic variant of Castleman disease.

**Immunohistochemistry-** CD-20, CD-10, BcL-2 confirmed the reactive follicular architecture. CD-3 & CD-13 B highlighted the predominance of T-lymphoid and plasma cells respectively in the interfollicular zones. CD-23 highlighted the follicular dendritic network within germinal centres. Opinion: Plasma cell variant of Castleman disease with no features of malignancy identified.

**Fig. 4:**
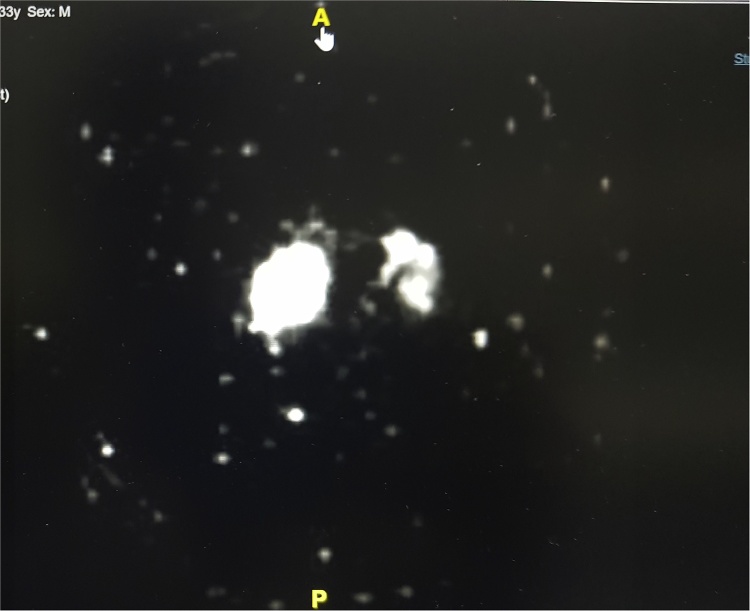
18 FDG PET-CT (Non-contrast) showing metabolically active left internal and external iliac lymph nodes.

**Fig. 5:**
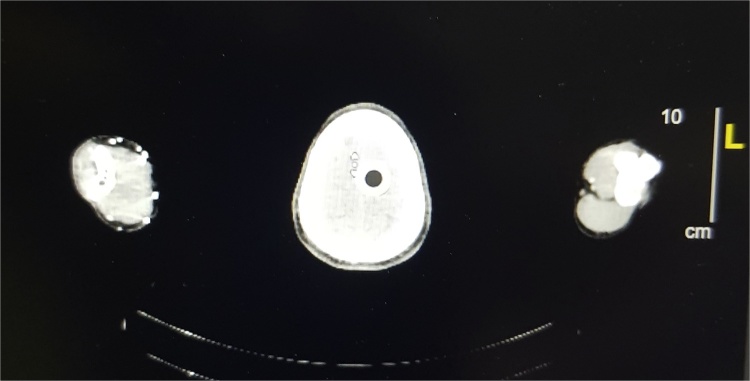
18 FDG PET-CT (Non-contrast) showing metabolically active left axillary lymph nodes.

**18 FDG PET-CT (Non-contrast):** Metabolically active left internal iliac, external iliac (Fig. 4), left inguinal, bilateral cervical, left axillary lymph nodes (Fig. 5) were noted.

## 3 Laboratory investigations

Hemogram: Hemoglobin 12 g/ dL, blood smear suggestive of microcytic hypochromic anemia; platelet count- normal.

Serum LDH- Normal; C-Reactive Protein- Normal; ESR 30 mm/1h

Liver and renal function tests- normal.

Viral serology-

 Hepatitis-B surface antigen (HBsAg) - Negative

 Hepatitis-C antibodies (Anti HCV) - Negative

 HIV antibodies- Nonreactive

Plasma PCR for HHV8- Negative

Investigations for Tuberculosis- Negative

Autoimmune disorders workup -Negative

Serum electrophoresis:

• Abnormal, suggestive of polyclonal gammopathy.
• Hyper Gamma Globulin region seen with IgG-1700 mg/dL
• Kappa-1680 mg/dL; Lambda-730 mg/dL.

Our patient fulfilled both major criteria; 3 laboratory criteria and 1 clinical criteria for IMCCD (Table 1).

**Table 1:** Diagnostic criteria for IMCCD: (Adapted from Fajgenbaum et al: International, evidence-based consensus diagnostic criteria for HHV-8-negative/idiopathic multicentric Castleman disease) [3].

Major Criteria:	
	1 Histopathology of the lymph node consistent with CD 2 Enlarged lymph nodes (>1 cm in short axis) in >2 lymph node stations
Minor Criteria:	
	A.) Laboratory: 1 Elevated CRP (>10 mg /L) or ESR > 15 mm/Hour 2 Anemia (<12.5 g/dl in males, <11.5 g/ dL in females) 3 Thrombocytopenia (Platelet count <150,000 /mm^3^) or thrombocytosis (Platelet count >400,000/mm^3^) 4 Hypoalbuminemia (Serum albumin <3.5 g/ dL) 5 Renal dysfunction (eGFR <60 ml/min /1.73 m^2^) or proteinuria (total protein 150 mg/24 h or 10 mg/dL) 6 Polyclonal hypergammaglobulinemia (total gamma globulin or IgG >1700 mg/dL) B.) Clinical: 1 Constitutional symptoms: night sweats, fever >38 °C, weight loss, fatigue. 2 Splenomegaly and/or hepatomegaly 3 Fluid accumulation: edema, anasarca, ascites, pleural effusion 4 Eruptive cherry hemangiomatosis or violaceous papules 5 Lymphocytic interstitial pneumonitis

^*^For diagnosis of IMCCD both major criteria and at least 2 minor criteria of which at least one is laboratory abnormality should be present after excluding infection related disorders, autoimmune/ autoinflammatory diseases and malignant/ lymphoproliferative disorders.

Patient was started on oral corticosteroids. In view of worsening systemic symptoms, increase in lymph node size and lower limb edema patient was subsequently started on IV Rituximab.

## 4 Discussion

Castleman disease (CD) is a rare condition of lymphoreticular system described by Benjamin Castleman in 1956.

### 4.1 Pathology and aetiopathogeneis of CD

CD is classified into 4 recognizable histopathological variants: [4,6]

I) Hyaline vascular variant: shows hyalinized vessels penetrating atrophic germinal centres with concentric rings of small lymphocytes widening the mantle zone (onion skin appearance). Germinal centres may have dysplastic follicular dendritic cells.
II) Plasma cell variant: shows hyperplastic germinal centres with interfollicular polyclonal plasmacytosis.
III) Mixed variant.
IV) Plasmablastic- exclusively seen in HSSV-8 associated MCCD.

Pathology features in CD are considered to be reactive to a state of increased cytokines including Interleukin-6 (IL-6) [6]. Proinflammatory state is common to both IMCCD and HSSV-8 associated MCCD, though the aetiology is different [6]. IMCCD could be due to autoantibodies or germline mutations involving immune system (autoimmune/ autoinflammatory hypothesis), malignancy (paraneoplastic hypothesis) or due to an unknown virus (viral hypothesis) [3]. HSSV-8 encodes a number of proteins including those involved in cytokine signaling. Viral encoded IL-6 shares 25% homology with human IL-6 and can trigger cytokine pathway leading to CD manifestations [4].

The pathology in Case-1 was hyaline vascular variant of CD and in Case-2, it was plasmacytic variant.

Clinically CD is classified into: [3,4]

a Unicentric Castleman Disease (UCCD)
b HHV8 associated Multicentric Castleman Disease (MCCD-H)
c Idiopathic Multicentric Castleman Disease (IMCCD)

UCCD: It accounts for 70% of cases of CD. Clinically it is a distinct entity producing an indolent localized disease with involvement of a single group of lymph nodes. Systemic manifestations are absent or minimal. The disease may be diagnosed incidentally on imaging or detected while investigating for pressure symptoms caused by the lymph node mass on adjacent structures.

In Case-1, our patient had presented with right iliac lymphadenopathy with pressure symptoms on the right femoral nerve. Investigations proved unicentricity of the disease.

MCCD: It is a serious systemic disease often presenting with involvement of multiple lymph node stations. Constitutional symptoms like fever, weight loss and debility often occur. It can progress and become lethal due to: exaggerated systemic inflammatory response and multi-organ dysfunction caused by “Cytokine storm” often involving IL-6 [3]. Additionally immunosuppression can occur due to cytopenias or polyclonal lymphoproliferation as also malignant transformation. Due to increased expression of cytokine pathways, patient may have “capillary leak syndrome” leading to anasarca, cardiac failure, renal failure, interstitial lung disease or liver failure. Thrombocytosis or thrombcytopenia, polyclonal gammopathy, increased inflammatory markers portend poor prognosis [7].

Human Herpes Simplex Virus 8 (HHV8) associated MCCD is a very aggressive disease [8] occurring in immunocompromised individuals due to the viral trigger and constitutes 65% of MCCD.

Idiopathic MCCD (IMCCD) has no known biomarker [3] and is diagnosed after excluding infective, autoimmune and malignant conditions of lymphoid tissue [3]. TAFRO syndrome (Thrombocytopenia, anasarca, myelofibrosis, renal dysfunction and organomegaly) is a subset of IMCCD and is a highly aggressive phenotype [9]. Hyaline vascular variant is the common pathology. It has a worse outcome as compared to Non-TAFRO IMCCD due to capillary leak syndrome [9].

The diagnosis of IMCCD is complex as it is evident from the work-up that was required for our Case-2. This patient fulfilled both the major criteria and 4 minor criteria for IMCCD (increased ESR, anemia, polyclonal gammopathy and constitutional symptoms).

Surgery is the primary recommended treatment for UCCD and has good long term prognosis with near normal survival [10]. Unresectable tumors can be treated with external beam radiotherapy or systemic treatment [11]. In Case-1, the patient is well 6 months after surgery.

MCCD is a more aggressive disease with 65% average 5-year survival [3]. There are no firm recommendations for MCCD at present due to the rarity and heterogeneous nature of the disease [4]. Many agents have been tried and recommended. In HSSV-8 associated MCCD, antiviral therapy is effective by decreasing the viral load and downregulating the cytokine pathway. ^4^Highly active anti-retroviral therapy (HAART) improves survival in HSSV-8 associated MCCD with HIV co-infection [4]. Immunosuppressants should be used with caution in the presence of active viral disease. In IMCCD corticosteroids have been used to palliate symptoms before starting aggressive systemic chemotherapy. Monoclonal antibodies targeting CD-20 on B-cells (Rituximab) have been tried as a single agent as well as in combination with chemotherapy [4]. Targeted therapy against IL-6 is very effective and well tolerated. Siltuximab is FDA approved, but is costly and not freely available and requires long-term administration [12,13].

Our Case-2 patient was started on corticosteroids initially and subsequently Rituximab was added on disease progression.

These two cases showcase the two ends of the clinical spectrum of CD requiring different management protocols. Awareness among surgeons and diligent work-up is imperative for early diagnosis and best outcome.

## 5 Conclusion

• CD is a rare heterogeneous group of hyperimmune lymphoproliferative disorders with a wide spectrum of clinical presentation requiring appropriate management strategies.
• UCCD is primarily treated by surgical excision with good outcome.
• MCCD is a systemic disease and requires systemic interventions.
• Primary treatment of HSSV-8 associated MCCD is aimed at decreasing the viral load.
• IMCCD is a diagnosis of exclusion and requires systemic treatment. IL-6 blockade gives encouraging results.

## Conflicts of interest

No conflict of interest.

## Sources of funding

No external research funding was received. The reported patients were treated free in the Government hospital.

## Ethical approval

Both the patients reported were treated according to accepted and published medical guidelines. No trial of any nature was done. Written and informed consent of both the patients has been taken for publication of the findings.

## Consent

Written and informed consent of both the patients has been taken for publication of the findings.

Patient’s identity is not being revealed in any manner in the report by way of images or personal identification details.

## Author contribution

Contribution of First Author, Corresponding author: Dr.Radhika Raj C. Govindaraju

1 Study Concept and design.
2 Analysis and interpretation of data.
3 Drafting of manuscript.
4 Critical revision.

Contribution of Second Author Dr. Suresh B

1 Acquisition of data.
2 Analysis and interpretation of data.

## Registration of research studies

Research Registry UIN: researchregistry4449.

## Guarantor

Dr. Radhika Raj C Govindaraju,

Associate Professor of Surgery,

Goa Medical College, Bambolim, Goa. INDIA-403202.

## Provenance and peer review

Not commissioned, externally peer reviewed.
